# Call combinations in birds and the evolution of compositional syntax

**DOI:** 10.1371/journal.pbio.2006532

**Published:** 2018-08-15

**Authors:** Toshitaka N. Suzuki, David Wheatcroft, Michael Griesser

**Affiliations:** 1 Department of Evolutionary Studies of Biosystems, SOKENDAI (The Graduate University for Advanced Studies), Kanagawa, Japan; 2 Department of Animal Ecology, Uppsala University, Uppsala, Sweden; 3 Department of Evolutionary Biology and Environmental Studies, University of Zurich, Zurich, Switzerland

## Abstract

Syntax is the set of rules for combining words into phrases, providing the basis for the generative power of linguistic expressions. In human language, the principle of compositionality governs how words are combined into a larger unit, the meaning of which depends on both the meanings of the words and the way in which they are combined. This linguistic capability, i.e., compositional syntax, has long been considered a trait unique to human language. Here, we review recent studies on call combinations in a passerine bird, the Japanese tit (*Parus minor*), that provide the first firm evidence for compositional syntax in a nonhuman animal. While it has been suggested that the findings of these studies fail to provide evidence for compositionality in Japanese tits, this criticism is based on misunderstanding of experimental design, misrepresentation of the importance of word order in human syntax, and necessitating linguistic capabilities beyond those given by the standard definition of compositionality. We argue that research on avian call combinations has provided the first steps in elucidating how compositional expressions could have emerged in animal communication systems.

Language is an essential human trait, but understanding its evolution remains a challenge, in large part because none of humans’ close evolutionary relatives (i.e., the great apes) possess communication systems approaching the complexity and flexibility of language [1]. The communicative power of language depends on multiple co-occurring capabilities, including referentiality, compositionality, syntax, recursivity, and vocal learning [2]. Thus, a profitable approach to gain insights into language evolution is to find and use appropriate analogies of these capabilities in other animals for comparative studies [1,2]. Over the past three decades, both field and laboratory studies have revealed that several linguistic capabilities, such as vocal learning and referentiality, did evolve in a range of animal taxa, providing insights into possible evolutionary drivers of these faculties of language (in a broad sense) [2]. Here, we review recent studies on bird calls demonstrating the first evidence for another key linguistic capability (i.e., ‘compositional syntax’ [3]) in a nonhuman vocal system [4–6] and discuss recent criticism of the interpretation of these studies.

Syntax is the set of rules for combining words into phrases. In human syntax, combinations of words follow ‘the principle of compositionality’, in which the meaning of a combination depends on both the meaning of its parts and the way in which they are combined [3]. Although combinations of meaningful calls have been documented for nonhuman primates [7,8], they have typically been considered noncompositional, i.e., a structured sequence provides an independent meaning as a whole [3,9]. The lack of evidence for compositional syntax in nonhuman animals makes it difficult to study its origins and evolution using comparative approaches. Assigning meaning to animal signals in the way in which we would assign meaning to human words is a challenge, but the meaning of animal signals could be assessed by observing and associating responses to different types of signals. Thus, three conditions are necessary to demonstrate compositional syntax in nonhuman animals: (i) production of call sequences depends on the context; (ii) response to the whole sequence depends on assessing the meanings of the component calls, rather than simply responding to the whole sequence as a single meaningful unit; and (iii) response to the whole sequence depends on the way in which the component calls are combined.

Recent studies have revealed that call combinations in a bird species, the Japanese tit (*Parus minor*) (Fig 1A), satisfy all three criteria for compositional syntax. Japanese tits have over 10 different notes in their vocal repertoire and use them either singly or in combination with other notes [4]. They produce ‘ABC’ calls when warning conspecifics about predators, while they produce ‘D’ calls when attracting conspecifics [4,5]. Interestingly, tits combine these two calls into ‘ABC-D’ sequences when recruiting conspecifics to mob a stationary predator [4]. Playback experiments [5] showed that tits display different behaviours when hearing ABC calls (moving their heads horizontally as if scanning for danger) and D calls (approaching the sound source). In response to ABC-D sequences, they produce both behaviours. Importantly, tits do not first scan and then approach, as would be predicted if they perceived ABC-D sequences as linear, ordered strings. Instead, they progressively approach the sound source while continuously scanning (Fig 1B). In contrast, playback of artificially reversed sequences (‘D-ABC’; Fig 1B) elicited reduced scanning and approaching compared to the response towards natural call sequences (ABC-D). Finally, we addressed the possibility that receivers simply associate the whole ABC-D sequence with a single ‘mobbing’ meaning rather than assessing the meanings of the constituent calls. We substituted willow tit ‘tää’ calls, which evoke similar responses in Japanese tits to their own D calls [10], into novel ABC-tää and reversed tää-ABC sequences [6]. We found that Japanese tits exhibit an equivalent response (i.e., approach with scanning) to both natural (ABC-D) and novel (ABC-tää) call sequences. In contrast, when these calls are combined into reversed ordering (tää-ABC), they reduced their responses. Together, these results demonstrate that tits combine different meaningful signals to generate a compound message that depends on the meaning of the elements and the way in which they are combined, i.e., compositional syntax [3]. Thus, we argue that changing the order in which the calls are combined does not produce an alternative compound message but rather a sequence with unclear or ambiguous meaning.

**Fig 1:**
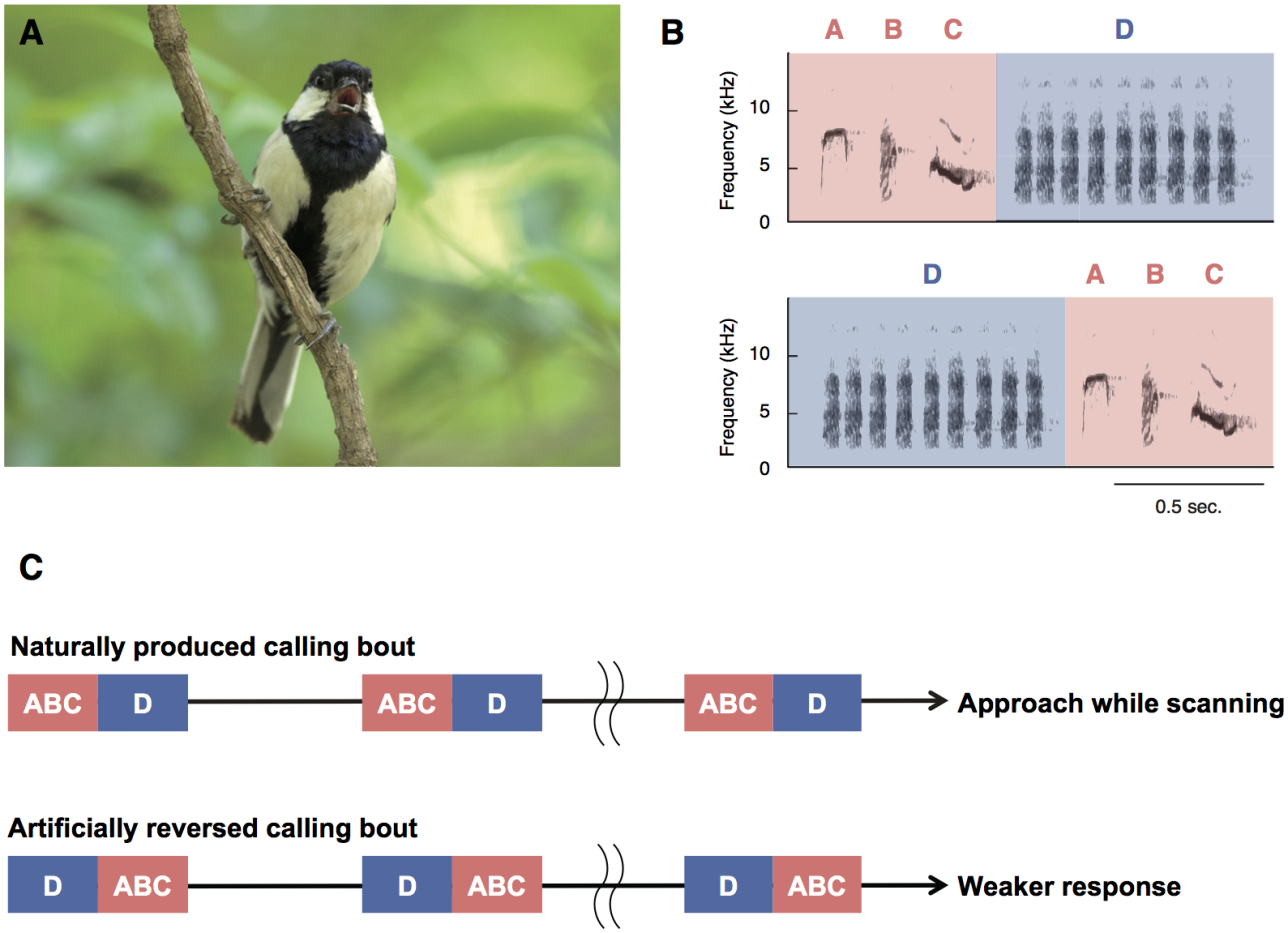
Schematic representation of experimental design. (**A**) Japanese tit. (**B**) Sound spectrogram of naturally produced ABC-D and artificially reversed D-ABC sequence. (**C**) Schematic representation of playback stimulus and responses (Experiment 2 in [5]). A different response to ABC-D and D-ABC sequences indicates that tits recognize call ordering when decoding the sequences. *Photo credit*: *Toshitaka N*. *Suzuki*.

Bolhuis and colleagues [11] argue that these findings do not provide evidence for compositional syntax. They make three main arguments: (i) that tits’ lack of responses to D-ABC combinations are a result of such messages being maladaptive; (ii) that linear order plays an unimportant role in human syntax; and (iii) that tit call combinations are not hierarchical or freely productive, as are combinations in human language.

First, they suggest that tits produce weaker responses to reversed (D-ABC) order sequences because only scanning (ABC) before approaching (D), but not the reversed response, would be adaptive. This interpretation, however, is likely to be based on misunderstanding the experimental set-up and tits’ behavioural responses. Tits naturally combine ABC and D calls with a 0.5- to 0.15-s interval in between the two calls [5]. This close temporal proximity does not allow tits to display an independent response (scan or approach) to the first call part (ABC or D) before hearing the second call part (D or ABC) (Fig 1B). Moreover, calls were not broadcast in isolation (i.e., a single ABC-D call) but rather in a calling bout, in which tens of sequences were produced with gaps of at least 1.6 s in between. Accordingly, tits do not exhibit a defined sequence of response to each call sequence (e.g., scan and then approach), but exhibit a compound response (approach while scanning) in response to playback of ABC-D calling bouts (Fig 1C). Thus, independent of the adaptiveness of responses, the fact that tits responded differently to natural (ABC-D) and reversed (D-ABC) sequences demonstrated that they perceived the order of call elements when decoding call sequences.

The difference in the tits’ responses to naturally produced ABC-D and artificially reversed D-ABC sequences implies that meaning in call sequences is dependent on the order of the lexical elements. However, another argument of Bolhuis and colleagues [11] is that linear order does not play as important a role in human language as is typically thought, and thus, differential responses to ABC-D and D-ABC sequences cannot provide evidence for compositional syntax. This claim is based on the example of the noun phrase ‘old men and women’. It can be interpreted as either a collection of old men and women of unspecified age or a collection of old men and old women (Fig 2A). Clearly, structural ambiguity of linguistic expressions can only be observed when more than two morphemes are strung together and are most easily recognized in written language, a highly derived form of language. A phrase with two words usually does have an unambiguous meaning, and in many cases, word order matters when producing a compound meaning. E.g., in English, ‘watch out’ has an unambiguous meaning, while the reversed combination ‘out watch’ is difficult to parse and has ambiguous, unclear meaning (Fig 2B). We would predict human responders to respond to the first phrase but not the second precisely because it violates syntactical rules. This is exactly what we observed in Japanese tits: combinations of ABC and D calls evoke compound responses only when they are ordered as ABC-D sequences (Fig 2C).

**Fig 2:**
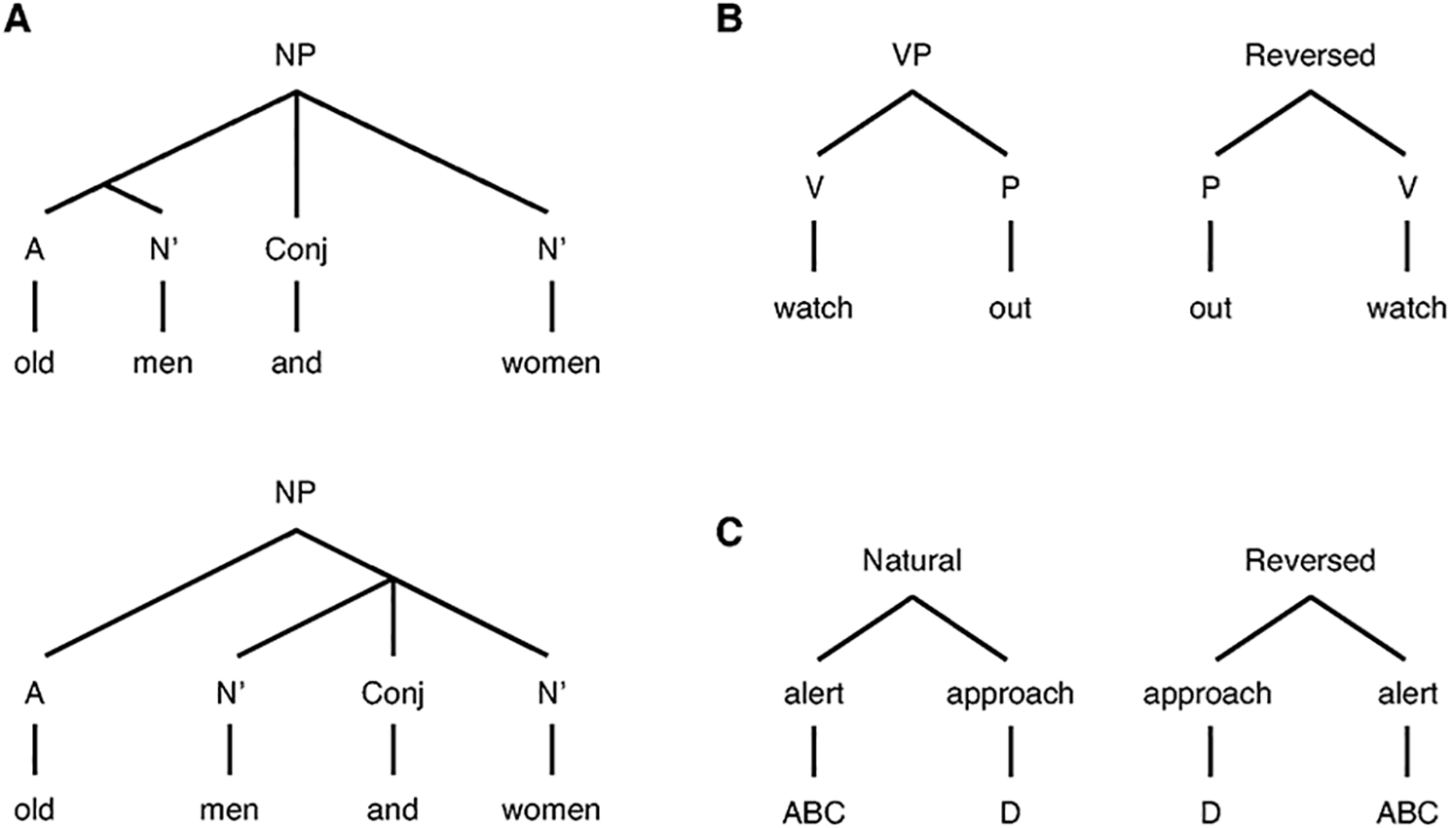
Examples of parse trees for human phrases and Japanese tit call sequences. (**A**) Ambiguity of meaning can be observed when more than two words are strung together ([[old men] and [women]] or [old [men and women]], see [11]). (**B**) The meaning of phrase depends on syntactic structure. (**C**) The meaning of Japanese tit sequence also depends on how meaningful calls are combined, indicating that there is also a syntactic structure in which call ordering governs the meaning of sequence. A, adjective; Conj, conjunctive; N’, noun; NP, noun phrase; P, preposition; V, verb; VP, verb phrase.

Finally, Bolhuis and colleagues [11] argue that hierarchical structure and free productivity are essential components of human language that are absent in tit calls. We agree that neither of these components have been demonstrated in tit call combinations, but neither are they part of even Bolhuis and colleagues’ [11] own preferred definition of compositionality in human language. Arguing that these features are not present in Japanese tit call sequences is not suitable to reject the interpretation of our findings. We note that parallels between vocal learning of birdsongs and human language are commonly made [12,13]. An important component of human language learning, unlike birdsong learning, is that meanings are associated to signals [3]. One could argue, as Bolhuis and colleagues [11] do about Japanese tit call combinations, that because this component of human language is absent in birdsong learning, any comparison between the two processes is invalid. Instead, we recognize that previous and ongoing work into birdsong has indeed uncovered deep insight into the neural, genomic, behavioural basis, and evolution of vocal learning more generally, despite the differences between birds and humans. Likewise, we are confident that future research into animal call combinations will provide similar insights into the evolution of compositionality.

In summary, we assert that the comments by Bolhuis and colleagues [11] do not change any interpretation of our results or the conclusion of the studies [4–6]. Our research, and that of others, has shown that call combinations incorporate different meaningful elements, which provides a new model to study the syntax–semantics link in nonhuman species [14]. A growing body of research reveals that combinations of meaningful calls have evolved in nonhuman animals such as Campbell’s monkeys (*Cercopithecus campbelli*) [8] and pied babblers (*Turdoides bicolor*) [15], suggesting that compositional syntax may have evolved independently in multiple animal taxa [16]. In addition, recent studies have shown that Japanese tits communicate about predatory threats by using combinations of different linguistic capabilities, such as referential signals and compositional syntax [4,17]. In light of our findings, we believe that animal call sequences can provide an invaluable model system for comparative studies to uncover the origins and evolution of linguistic capabilities that form the basis of syntactic communication.

## Abbreviations

A: adjective
Conj: conjunctive
N’: noun
NP: noun phrase
P: preposition
V: verb
VP: verb phrase
